# Establishment of a mathematical prediction model for voriconazole stable maintenance dose: a prospective study

**DOI:** 10.3389/fcimb.2023.1157944

**Published:** 2023-07-26

**Authors:** Lijuan Zhou, Min Li, Huihong Li, Zhiqiang Guo, Yanqiu Gao, Hua Zhang, Fuli Qin, Zhihui Sang, Qinghe Xing, Long Cheng, Wei Cao

**Affiliations:** ^1^ Translational Medicine Center, Zhengzhou Central Hospital Affiliated to Zhengzhou University, Zhengzhou, Henan, China; ^2^ Department of Hematology, Zhengzhou Central Hospital Affiliated to Zhengzhou University, Zhengzhou, Henan, China; ^3^ Department of Respiratory Medicine, Zhengzhou Central Hospital Affiliated to Zhengzhou University, Zhengzhou, Henan, China; ^4^ College of Pharmacy, Xinxiang Medical University, Xinxiang, Henan, China; ^5^ Institutes of Biomedical Sciences and Children’s Hospital, Fudan University, Shanghai, China; ^6^ College of Nursing, Chifeng University, Chifeng, Inner Mongolia, China

**Keywords:** voriconazole, CYP2C19, prediction model, proinflammatory cytokines, security

## Abstract

**Background:**

In patients with invasive fungal infection (IFI), the steady-state serum trough concentration (*C*_min_) of voriconazole (VCZ) is highly variable and can lead to treatment failure (*C*_min_ < 0.5 mg/L) and toxicity (*C*_min_ ≥ 5.0 mg/L). However, It remains challenging to determine the ideal maintenance dose to achieve the desired *C*_min_ level quickly.

**Aims:**

This randomized, prospective observational single-center study aimed to identify factors affecting VCZ-*C*_min_ and maintenance dose and create an algorithmic model to predict the necessary maintenance dose. MeThe study enrolled 306 adult IFI patients, split into two groups: non-gene-directed (A) (where CYP2C19 phenotype is not involved in determining VCZ dose) and gene-directed (B) (where CYP2C19 phenotype is involved in determining VCZ dose).

**Results:**

Results indicated that CYP2C19 genetic polymorphisms might significantly impact VCZ loading and maintenance dose selection. CYP2C19 phenotype, C-reaction protein (CRP), and average daily dose/body weight were significant influencers on VCZ-*C*_min_, while CYP2C19 phenotype, CRP, and body weight significantly impacted VCZ maintenance dose. A feasible predictive formula for VCZ stable maintenance dose was derived from the regression equation as a maintenance dose (mg) =282.774-0.735×age (year)+2.946×body weight(Kg)-19.402×CYP2C19 phenotype (UM/RM/NM:0, IM:1, PM:2)-0.316×CRP (mg/L) (*p* < 0.001).

**Discussion:**

DiThis formula may serve as a valuable supplement to the Clinical Pharmacogenetics Implementation Consortium (CPIC^®^) guideline for CYP2C19 and VCZ therapy, especially for IFI patients with highly variable inflammatory cytokines during VCZ therapy.

## Introduction

Despite medical advances, fungal infections are a significant morbidity and mortality cause. Invasive fungal infection (IFI) in critically ill patients continues to present a hurdle and significant challenge for the clinician (Bassetti et al., 2017; Yan et al., 2018; Resendiz-Sharpe et al., 2019). Voriconazole (VCZ), a preferred first-line broad-spectrum triazole antifungal agent, is widely used to treat IFI (Limper et al., 2011; Lortholary et al., 2012; Ullmann et al., 2012), including pathogens of Aspergillus and Candida species, as well as other mold infections (Tissot et al., 2017; Lestrade et al., 2019). Compared with fluconazole, VCZ provides an improved therapeutic option for treating life-threatening fungal infections (Peman et al., 2006).

However, individualized dose adjustment is critical to achieving optimal serum VCZ concentration. VCZ has non-linear pharmacokinetics due to its saturable metabolism. The proportion of exposure dose increase is much more significant than the dose increase for VCZ. Therefore, VCZ serum trough concentrations (VCZ-*C*_min_) are highly variable in clinical practice, with variability observed among patients and the same patients over time (Schulz et al., 2019). These variability factors are complex and include age, body weight, obesity, baseline status of patients, liver functions, polymorphisms of drug-metabolizing enzymes, drug interactions, and inflammatory factors such as C-reaction protein (CRP), Interleukin-6 (IL-6) and IL-1β (Koselke et al., 2012; Dolton et al., 2014; Valle-T-Figueras et al., 2021; Aiuchi et al., 2022; Li et al., 2022). Many studies and our previous studies have suggested that inflammatory factors [CRP, IL-6, IL-1, and procalcitonina (PCT)] can significantly affect the pharmacokinetics of VCZ, resulting in an increase in VCZ-*C*_min_ in patients (Shah and Smith, 2015; Zeng et al., 2020; Darakjian et al., 2021). However, subtherapeutic and supratherapeutic concentrations of VCZ are associated with increased mortality in patients with IFI and a high frequency of adverse events such as hepatotoxicity, neurotoxicity, and visual disorders, respectively (Farkas et al., 2016). Therapeutic drug monitoring (TDM) can be widely used to assess the efficacy and safety of VCZ (Richards et al., 2017; Takesue et al., 2022). To decrease the risk of therapeutic failure and drug-related toxicity, the Chinese Pharmacological Society has recommended a reasonable VCZ-*C*_min_ range of 0.5-5.0 mg/L for efficacy and safety in the Chinese population (Jin et al., 2016; Chen et al., 2018; Mafuru et al., 2019). Current research mainly focuses on finding the factors that influence the variation of VCZ-*C*_min_. The highly variable VCZ-*C*_min_ caused by the non-linear pharmacokinetics poses a significant challenge to optimal dosing. It is worth considering how to personalize the VCZ maintenance dose to achieve an effective *C*_min_.

The high interindividual pharmacokinetic variability of VCZ is mainly influenced by the liver through cytochrome CYP2C19 and, to a lesser extent, by CYP2C9 and CYP3A4 (Xu et al., 2018; Kim et al., 2019). VCZ-*C*_min_ in CYP2C19 intermediate and poor metabolizers is found to be 1.64 and 2.61 times higher compared to normal metabolizers (Miao et al., 2019). The Clinical Pharmacogenetics Implementation Consortium (CPIC^®^) guideline summarizes evidence from the literature supporting this association. It provides therapeutic recommendations for using VCZ for treatment based on the CYP2C19 genotype, published in Clinical Pharmacology and Therapeutics in 2017 (Moriyama et al., 2017). However, the evidence is still insufficient regarding the relationship between CYP2C19 genetic status and loading dose for VCZ. Formulating a multivariate regression of these related-*C*_min_ factors into the stable maintenance dose formula is still a challenge.

Therefore, the study aimed to (і) Determine the guiding significance of the CYP2C19 genetic polymorphisms for the initial loading dose of VCZ in ill patients; (ii) Determine factors affecting VCZ-*C*_min_ and maintenance dose; (iii) Establish the prediction model formula for a stable maintenance dose of VCZ.

## Materials and methods

### Standard protocol approvals, registrations and patient consents

**Figure 1:**
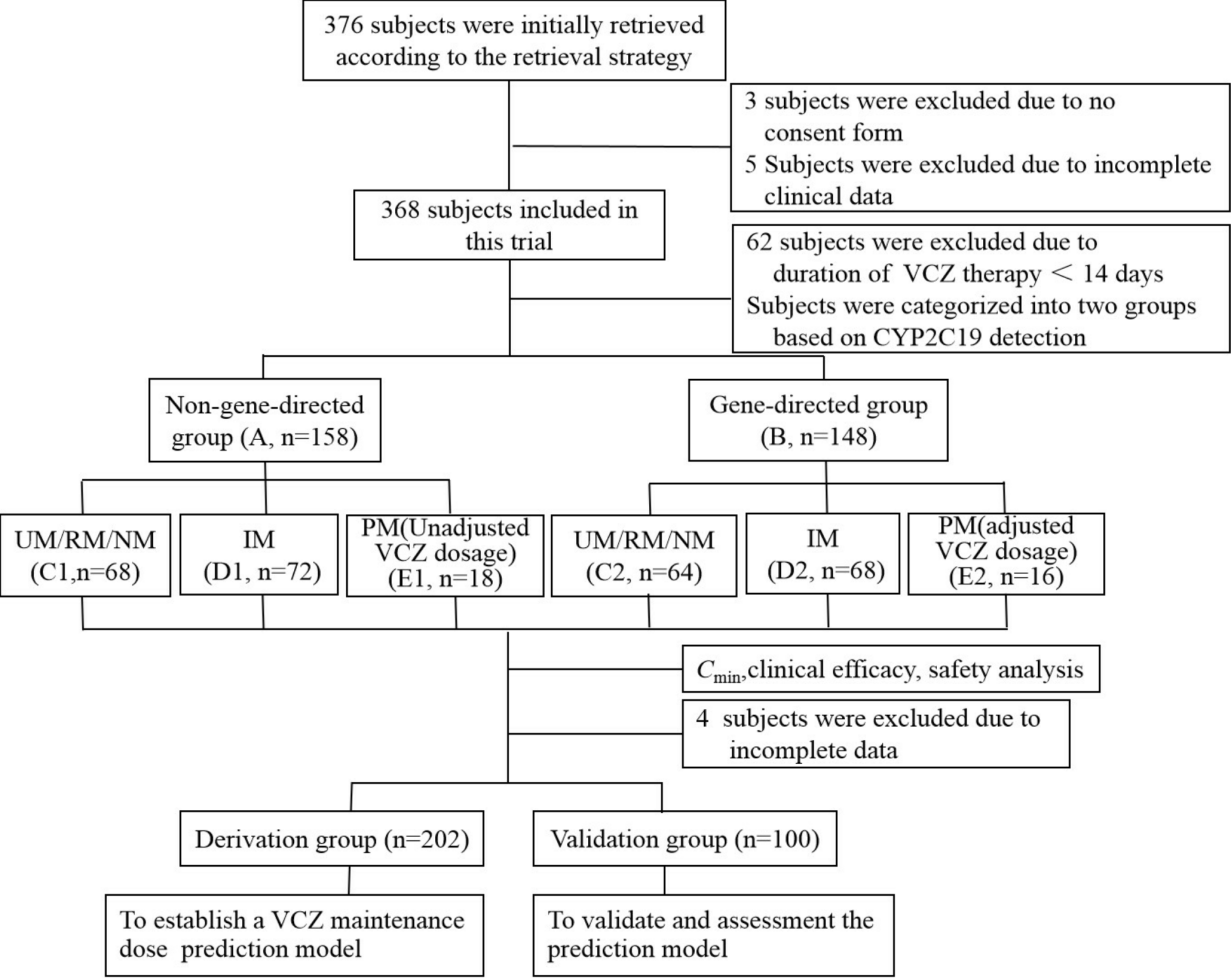
Study flow diagram. VCZ, voriconazole; *C*_min_, serum trough concentration. UM, Ultrarapid metabolizer; RM, rapid metabolizer; NM, normal metabolizer; IM, intermediate metabolizer; PM, poor metabolizer.

A prospective cohort study was conducted at Zhengzhou Central Hospital Affiliated to Zhengzhou University from August 2018 to August 2021. Hospitalized patients who met the following inclusion criteria were eligible for the study: (і) age ≥ 18 years old; (ii) diagnosed with invasive fungal infection according to the criteria established by De Pauw et al. (De Pauw et al., 2008); (iii) received VCZ therapy for ≥ 14 days; and (iv) had accurate, complete, and available efficacy and safety data. The exclusion criteria were: (і) patients allergic to VCZ or with poor compliance; (ii) use of other antifungal drugs during VCZ treatment; (iii) not eligible for blood sampling monitored by blood concentration; (iv) patients with severe liver function impairment (alanine aminotransferase (ALT) and aspartate aminotransferase (AST) before VCZ treatment are more significant than 3 times the standard upper limit, total bilirubin (TBIL) is greater than 2 times the standard upper limit); (v) pregnant or lactating women; and (vi) those who had participated in other clinical trials in the past three months. A total of 306 patients were screened for the study (**Figure 1**). This study was conducted following the Declaration of Helsinki, and it was approved by the Investigational Review Board at Zhengzhou Central Hospital Affiliated to Zhengzhou University (No:201973). The clinical trials were registered in the clinical trial registry at https://clinicaltrials.gov/ (NCT04004078). All patients or their legal representatives provided written informed consent.

### Treatment regimen and groups

The patients were randomly assigned to either the non-gene-directed group (Group A, *n*=158), where CYP2C19 phenotype was not considered in determining VCZ dose, or the gene-directed group (Group B, *n*=148), where CYP2C19 phenotype was involved in determining VCZ dose. Group A received VCZ doses according to the drug instructions. After VCZ treatment, the CYP2C19 genotype of group A was determined. The patients were categorized into ultrarapid metabolizer (UM)/rapid metabolizer (RM)/normal metabolizer (NM) group (C1), intermediate metabolizer (IM) group (D1), and poor metabolizer (PM) group (E1), prepare for the construction of mathematical models in the derivation group. Before VCZ administration, Group B patients were categorized into UM/RM/NM group (C2), IM group (D2), and PM group (E2) based on their CYP2C19 genotype. All the recruited patients were treated with VCZ for a duration of 14 to 93 days, and the dosage of the administration is outlined below. Group A received VCZ intravenously or orally 2 times at a loading dose of 450, 400, 350, or 300 mg at 12-hour intervals, followed by a maintenance dose of 350, 300, 250, or 200 mg at 12-hour intervals. For group B patients with RM, NM, and IM, the dosage of VCZ was the same as that of group A. For patients with PM, VCZ was intravenously or orally administered 2 times at a loading dose of 300 mg at 12-hour intervals, followed by a maintenance dose of 200 or 150 mg at 12-hour intervals. For the patients with UM, VCZ was administered at a dose that was 50% higher than the standard dose.

### CYP2C19 genotyping

CYP2C19 genotype was determined from peripheral blood, which was extracted and stored in an EDTA anticoagulant tube. Real-time fluorescence quantitative PCR (ThermoFisher Applied Biosystems 7500 fast PCR) was performed using a Human CYP2C19 gene detection kit (PCR-fluorescence probe method, Wuhan YZY Medical Science and Technology Co., Ltd, China) following the manufacturer’s instructions. Genomic DNA was isolated from whole blood using QIAamp DNA blood kits (Qiagen, Hilden, Germany). According to nomenclature by CPIC^®^, the CYP2C19 genotype was classified as ultrarapid metabolizer (*17/*17), rapid metabolizer (*1/*17), normal metabolizer (*1/*1), intermediate metabolizer (*1/*2, *1/*3, *2/*17, *3/*17), or poor metabolizer (*2/*2, *2/*3, *3/*3).

### Serum VCZ trough concentrations assay

The blood samples collected from the enrolled patients were centrifuged at 3500 r/min for 10 minutes. VCZ-*C*_min_ levels were measured at steady-state, 30 minutes before VCZ administration, using high-performance liquid chromatography with acetonitrile-water (53:47) as the mobile phase, a flow rate of 1.0 mL/min, and a wavelength of 256 nm. The *C*_min_ levels were monitored at least once for each patient. The Translational Medicine Center implemented TDM. The linearity range was 0.0635 ~ 21.16 mg/L (R^2 = ^0.9999), and the limit of quantitation and the detection limit were 0.8 ng and 0.3 ng, respectively. The intra-day and inter-day precisions were 1.37% and 1.86%, respectively. The average extraction recovery of VCZ was 98.03%, and the average method recovery was 99.52%, with an RSD of 0.83%.

### Cytokine concentrate assay

CRP, PCT, and IL-6 were detected using validated sandwich ELISA kits according to the manufacturer’s instructions at the Zhengzhou Clinical Laboratory Center. The interval for drawing blood between inflammatory factors and *C*_min_ is not more than 24 hours.

### Data collection

The hospital medical records of all patients included in the study were screened, reviewed, and analyzed by trained reviewers using a hospital information system. Clinical data were collected, including demographics, comorbidities, concomitant use of proton pump inhibitors (PPIs) and glucocorticoid, clinical information regarding VCZ dosing, duration of VCZ therapy, patients’ symptoms, body temperature, and chest X-Rays. Laboratory data included white blood cell counts, CRP, PCT, IL-6, gamma-glutamyl transpeptidase (GGT), AST, ALT, alkaline phosphatase (ALP), TBIL, albumin, blood urea nitrogen (BUN), serum creatinine (Scr), and Creatinine clearance.

### Efficacy assessment of VCZ

Patients who received VCZ for suspected invasive fungal infection (IFI) were classified according to the Invasive Fungal Infection Group criteria of the European Organization for Research and Treatment of Cancer and Mycoses Study Group of the National Institute of Allergy and Infectious Diseases. Efficacy assessment was performed by a team of three physicians led by the chief physician. The final clinical response to VCZ was evaluated based on the patients’ clinical symptoms, laboratory data, the Galactomannan experiment, 1,3-β-D-glucan experiment, bacteriological findings, and computed tomography. The response was classified as complete, partial, or treatment failure, with a complete response being defined as the resolution of signs and symptoms of the infectious process, including chest X-ray, and a partial response is defined as at least a 50% improvement in pulmonary infiltrates and signs and symptoms of the infection. Treatment failure was defined as the withdrawal of the treatment due to poor response, toxicity, or death associated with the infection (Ruiz et al., 2019).

### Safety assessment

Hepatotoxicity was defined as ALT or AST more than three times the upper limit of the institution’s normal reference ranges (ALT 0–40 U/L, AST 0–35 U/L), or TBil more than two times the upper limit of the institution’s normal reference ranges (TBIL 5.1–22 umol/L). For patients with abnormal values at baseline, hepatotoxicity was defined as ALT or AST more than three times or TBil more than two times the baseline value. The relationship between hepatotoxicity and VCZ was evaluated.

### Statistical analysis

Statistical analyses and randomization were performed using the Statistical Package for the Social Sciences software (ver.19.0; SPSS Inc.). Data were presented as the number of categorical variables or as mean ± standard deviation (mean ± SD) or median values (minimum-maximum) and interquartile range (IQR) for continuous variables. Chi-squared or Fisher’s exact test compared groups for categorical variables. Comparisons were conducted using the Student’s t-test or the Mann-Whitney U-test for continuous variables, one-way ANOVA analysis of variance, or the Kruskal-Wallis test. Pearson’s correlation analysis was used to analyze the relationship between inflammatory markers and *C*_min_. Multiple stepwise regression analyses were used to analyze significant influencing factors affecting VCZ-*C*_min_ and the VCZ maintenance dose. Multiple linear regression analyses established a VCZ stable maintenance dose prediction model. All tests were two-tailed, and a p-value of less than 0.05 was considered statistically significant.

## Results

### Baseline patient characteristics

The demographic and clinical characteristics of the included patients are presented in **Table 1**. The age of the studied patients ranged from 18 to 98 years. There were no significant differences in all indices between groups A and B, as well as between any two of all subgroups (group C1, C2, D1, D2, E1, and E2) (*p* > 0.05).

**Table 1:** Demographic and clinical characteristics of subjects in different groups.

Indexes	Non-gene-directed group (A) (N=158)	Gene-directed group (B) (N=148)	P value
Gender ^1)^ (*n*, %)			
Male	101(63.92)	89(60.14)	0.495
Female	57(36.08)	59(39.86)	
Age(years)^2)^	71.18 ± 16.20	71.22 ± 17.18	0.984
Body weight(kg)^a 2)^	62.54 ± 10.48	62.46 ± 11.40	0.943
Albumin(g/L)^a 2)^	30.93 ± 4.40	30.65 ± 4.69	0.588
ALT(U/L)^a 2)^	29.39 ± 18.99	28.42 ± 17.12	0.640
AST(U/L)^a 2)^	31.90 ± 15.43	32.69 ± 15.70	0.657
ALP(U/L)^a 2)^	93.80 ± 44.83	99.55 ± 39.26	0.235
GGT(U/L)^a 2)^	47.74 ± 40.69	49.49 ± 29.78	0.669
S_cr_(μmol/L)^a 2)^	66.11 ± 39.75	62.29 ± 34.56	0.371
BUN(mmol/L)^a 2)^	9.19 ± 6.54	9.38 ± 5.79	0.794
Co-medication (*n*, %)			
PPIs ^1)^	109(68.99)	111(75.00)	0.330
Glucocorticoid ^1)^	77(48.73)	76(51.35)	0.647
CRP ^a 2)^	68.25 ± 62.71	56.29 ± 58.56	0.086
CYP2C19 gene phenotype (*n*, %)			
UM/RM/NM ^1)^	68 (43.04)	58 (39.19)	0.971
IM^1)^	72 (45.57)	68 (45.95)	0.947
PM^1)^	18 (11.39)	16 (10.81)	0.871
Combined underlying diseases (*n*, %)			
Acute lymphocyte leukemia^1)^	31(19.62)	24(16.21)	0.438
Severe pneumonia^1)^	91(57.59)	96(64.86)	0.192
Respiratory Failure^1)^	64(40.51)	65(43.92)	0.546
Sepsis^1)^	43(27.22)	47(31.76)	0.384
Systemic Inflammatory ^1)^ Response Syndrome(SIRS)	11(6.96)	7(4.73)	0.407
Coronary heart disease ^1)^	56(35.44)	45(30.41)	0.349
Diabetes ^1)^	36(22.78)	27(18.24)	0.326
Hypertension ^1)^	54(34.18)	51(34.46)	0.959
Hyperlipemi ^1)^	36(22.78)	41(27.70)	0.322
Voriconazole route of administration(*n*, %)			
Intravenous ^1)^	121(76.58)	99(66.89)	0.059
Oral ^1)^	30(18.99)	37(25.00)	0.204
Sequential therapy ^1)^	7(4.43)	12(8.11)	0.183

Data are presented as the mean ± S.D. for continuous variables and as numbers for categorical variables.^a^ Values are reported for day 1 of voriconazole therapy. Statistical analyses were performed using either a chi-squared test or Student’s t-test. 1) A Chi-squared test 2) Student’s t-test. ALP, alkaline phosphatase; GGT, gamma-glutamyl transpeptidase; Scr, serum creatinine; BUN, blood urea nitrogen; CRP, C-reaction protein; Sequential therapy is defined as a method of continuing treatment with oral antibiotics that have a long half-life and bioavailability similar to that of injection, while switching between different dosage forms of the same drug.

### VCZ trough concentrations

**Figure 2:**
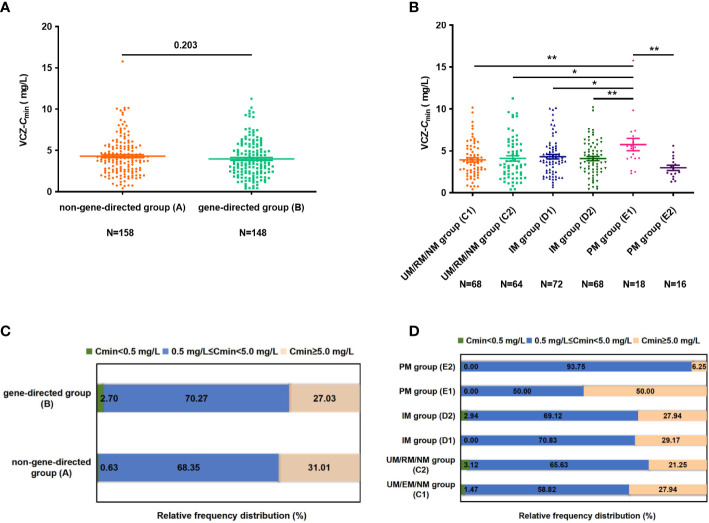
**(A)** The difference of VCZ-*C*_min_ between groups **(A, B)**. **(B)** The difference of VCZ-*C*_min_ among subgroups (group C1, C2, D1, D2, E1, and E2). **(C)** The percentage of patients obtaining *C*_min_ levels of < 0.5 mg/L, 0.5 mg/L ≤ *C*_min_ < 5.0 mg/L, and *C*_min_ > 5.0mg/L between groups **(A, B)**. **(D)** The percentage of patients achieving *C*_min_ level of < 0.5 mg/L, 0.5 mg/L ≤ *C*_min_ < 5.0 mg/L and *C*_min_ > 5.0 mg/L among subgroups (group C1, C2, D1, D2, E1, and E2). Data were expressed as mean ± SD and were analyzed using Student’s t-test. *p<0.05; **p<0.01.VCZ-*C*_min_, voriconazole serum trough concentration; UM, Ultrarapid metabolizer; RM, rapid metabolizer; NM, normal metabolizer; IM, intermediate metabolizer; PM, poor metabolizer.

A patient can undergo multiple *C*_min_ monitoring; 412 VCZ-*C*_min_ measurements were available from the included patient cohort. Of these, 306 initial VCZ-*C*_min_ measurements were available from each patient. Of the 412 concentrations, 370, 318, and 190 matched available CRP, PCT, and IL-6 determination samples. The VCZ-*C*_min_ was 3.97 ± 2.27 and 4.30 ± 2.36 mg/L in groups B and A, respectively (*p* = 0.203) (**Figure 2A**). The VCZ-*C*_min_ in group E1 was significantly higher than that in other groups (groups A, B, C1, C2, D1, D2, and E2, all *p* < 0.05), respectively (**Figures 2A, B**). That is to say, patients with different CYP2C19 genotypes were given the same dose of VCZ, while patients with the PM genotype can achieve higher VCZ-*C*_min_. This study found that VCZ-*C*_min_ for 0.63% and 31.01% of the patients in group A were subtherapeutic concentration (< 0.5 mg/L) and supratherapeutic concentration (≥ 5.0 mg/L), respectively. Notably, the proportion of therapeutic concentration (0.5-5.0 mg/L) was significantly higher in group E2 compared to group A (93.75% vs. 68.35%, *p* = 0.041) and group E1 (93.75% vs. 50.00%, *p* = 0.008) (**Figures 2C, D**).

### Correlations between Inflammatory factors and VCZ-*C*_min_

Twenty-eight out of 306 patients had complete indicators for VCZ-*C*_min_, CRP, PCT, and IL-6 measured simultaneously during their hospital stay. There was no dose adjustment for VCZ after each VCZ-*C*_min_ measurement for these 28 patients. Fourteen patients were measured twice, ten were measured three times, and four were measured four times. The changes in VCZ-*C*_min_, CRP, PCT, and IL-6 were consistent in 22 patients who were measured multiple times (**Supplementary Figures 1A-K, O-U, Y-AB**).

**Figure 3:**
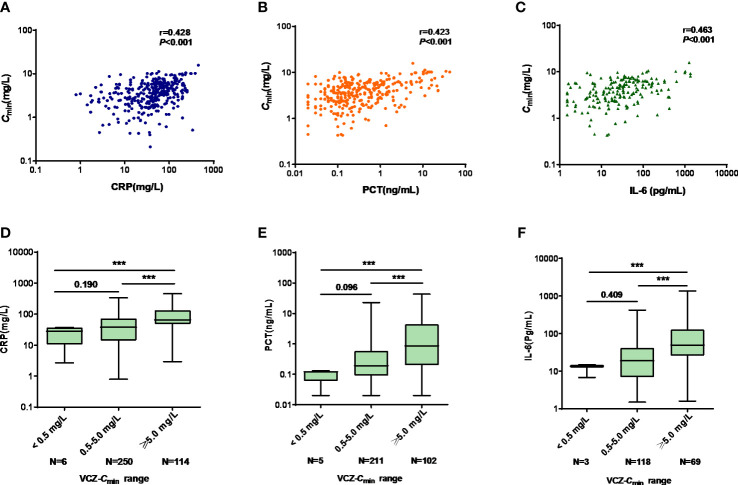
**(A–C)** The linear correlation between VCZ-*C*_min_ (mg/L) and CRP (mg/L), PCT (ng/mL), and IL-6 (pg/mL), respectively. **(A)** VCZ-*C*_min_ vs. CRP; **(B)** VCZ-*C*_min_ vs. PCT; **(C)** VCZ-*C*_min_ vs. IL-6. **(D–F)** Comparison of CRP, PCT, and IL-6 among *C*_min_ < 0.5 mg/L, 0.5 mg/L ≤ *C*_min_ < 5.0 mg/L, and *C*_min_ ≥ 5.0 mg/L. **(D)** CRP; **(E)** PCT; **(F)** IL-6. Data were expressed as the median values (range) and interquartile range (IQR) and were analyzed by Mann-Whitney U-test and Kruskal-Wallis test. ***p<0.001. VCZ-*C*_min_, voriconazole serum trough concentration; CRP, C-reactive protein; PCT, procalcitonin; IL-6, interleukin-6.

CRP, PCT, and IL-6 levels were significantly correlated with VCZ-*C*_min_ (r = 0.428, *p* < 0.001; r = 0.423, *p* < 0.001; r = 0.463, *p* < 0.001), respectively (**Figures 3A-C**).

There were significant differences in CRP, PCT, and IL-6 levels among patients with VCZ-*C*_min_ < 0.5 mg/L, 0.5 mg/L ≤ VCZ-*C*_min_<5.0 mg/L, and VCZ-*C*_min_ ≥ 5.0 mg/L, respectively (28.09 [2.68-37.30] vs. 38.04 [0.80-336.70] vs. 65.33 mg/L [2.94-453.00], *p* < 0.001; 0.12 [0.02-0.13] vs. 0.19 [0.02-22.84] vs. 0.86 ng/mL [0.02-43.00], *p* < 0.001; 13.60 [6.80-14.90] vs. 18.90 [1.50-423.10] vs. 49.60 pg/mL [1.60-1362.00], *p* < 0.001) (**Figures 3D-F**). The median concentrations of CRP, PCT, and IL-6 increased as the VCZ-*C*_min_ increased. VCZ-*C*_min_ ≥ 5.0 mg/L was predicted by CRP, PCT and IL-6, respectively. The cutoff values of CRP, PCT, and IL-6 were 48.82 mg/L, 0.995 ng/mL, and 28.50 pg/mL.

### *In vivo* impact of PPIs on the VCZ-*C*_min_

This study, 306 patients were divided into groups based on PPIs (esomeprazole, omeprazole, pantoprazole, rabeprazole) and a control group. The demographic and clinical characteristics of the included patients are displayed in **Table 2**. There were no significant differences in gender, age, body weight, albumin, ALT, AST, ALP, GGT, BUN, Scr, and CRP among the five groups or between any two of the five groups.

**Table 2:** Demographic and clinical characteristics of subjects in all groups.

Indexes	Control group (N=18)	Esomeprazole group (N=115)	Omeprazole group (N=31)	Rabeprazole group (N=22)	Pantoprazole group (N=20)	P value
Gender ^1)^ (Male/Female)	63/55	79/36	17/14	11/11	14/6	0.096
Age (years)^2)^	70.87 ± 17.01	74.85 ± 14.99	69.06 ± 14.64	69.64 ± 12.23	76.10 ± 18.78	0.141
Body weight (kg) ^a 2)^	61.87 ± 11.91	63.00 ± 11.62	59.55 ± 9.44	62.86 ± 12.43	63.60 ± 9.44	0.614
Albumin (g/L) ^a 3)^	30.60 [28.18, 34.82]	30.80 [28.70, 32.50]	30.00 [28.90, 34.50]	31.85 [28.68, 35.88]	30.40 [28.82, 32.75]	0.834
ALT (U/L) ^a 2)^	26.40 ± 19.04	29.52 ± 19.22	31.90 ± 21.39	26.27 ± 23.53	34.85 ± 25.39	0.322
AST (U/L) ^a 2)^	34.18 ± 15.81	38.47 ± 19.22	33.54 ± 21.79	33.68 ± 19.36	39.30 ± 20.13	0.310
ALP (U/L) ^a 2)^	105.27 ± 41.92	99.70 ± 50.46	109.97 ± 50.93	109.04 ± 38.71	97.35 ± 35.75	0.685
GGT (U/L) ^a 2)^	50.79 ± 40.67	45.33 ± 27.85	53.85 ± 35.44	49.50 ± 27.70	47.75 ± 35.64	0.711
BUN (mmol/L) ^a 2)^	9.11 ± 7.89	9.97 ± 6.14	7.81 ± 5.36	7.33 ± 4.28	10.58 ± 4.32	0.239
Scr (μmol/L) ^a 2)^	63.32 ± 39.85	74.78 ± 64.60	57.21 ± 32.71	74.55 ± 47.23	64.11 ± 22.00	0.279
CRP (mg/L) ^b 3)^	43.24 [18.67, 106.08]	45.90 [14.39, 73.24]	50.00 [16.42, 91.06]	42.11 [4.84, 107.56]	27.00 [8.80, 78.94]	0.717

Data are presented as the mean ± standard deviation or the medians (IQR) for continuous variables and the number for categorical variables.

a Values on day 1 of voriconazole therapy. The interval for drawing blood between inflammatory factors and VCZ-C_min_ is not more than 24 hours.

^1)^ Chi-squared test; ^2)^ One-way ANOVA analysis of variance; ^3)^ Kruskal-Wallis test. A Chi-squared test compared gender between two groups. Student’s t-test compared with age, body weight, ALT, AST, ALP, GGT, Scr, and BUN between two groups. The Mann-Whitney U-test compared albumin and CRP between the two groups. ALT, alanine aminotransferase; AST, aspartate aminotransferase; ALP, alkaline phosphatase; GGT, gamma-glutamyl transpeptidase; Scr, serum creatinine; BUN, blood urea nitrogen; CRP, C-reactive protein.

**Figure 4:**
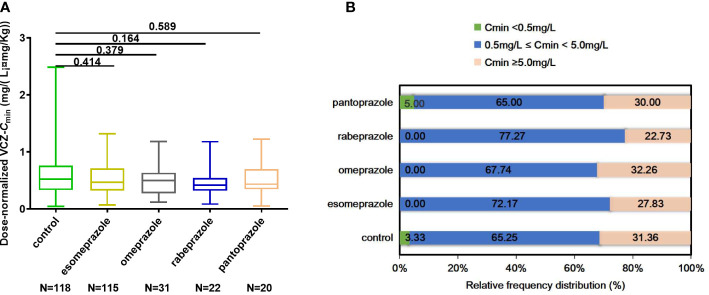
**(A)** Comparison of dose-normalized VCZ-*C*_min_ among control, esomeprazole, omeprazole, rabeprazole and pantoprazole groups. **(B)** The percentage of patients obtaining *C*_min_ < 0.5 mg/L, 0.5 mg/L ≤ *C*_min_ *<*5.0 mg/L, and *C*_min_ ≥ 5.0 mg/L among different groups. Data were expressed as mean ± SD and were analyzed by Student’s t-test. ***p<0.001. Dose-normalized VCZ-*C*_min_=[VCZ-*C*_min_ (mg/L)×body weight (Kg)]/average daily dose (mg); VCZ-*C*_min_,voriconazole serum trough concentration.

Each VCZ-*C*_min_ was adjusted according to body weight and daily dose to calculate dose-normalized VCZ-*C*_min_. It showed no significant difference in dose-normalized VCZ-*C*_min_ among the five groups (*p* = 0.584) (**Figure 4A**). The calculation for dose-normalized VCZ-*C*_min_ was as follows: Dose-normalized VCZ-*C*_min_=[VCZ-*C*_min_ (mg/L)×body weight (Kg)]/average daily dose (mg). There were no significant differences in the proportion of 0.5 mg/L ≤ *C*_min_<5.0 mg/L and *C*_min_ ≥ 5.0 mg/L among the five groups (*p* = 0.701; *p* = 0.775), respectively (**Figure 4B**).

### Clinical efficacy of VCZ and assessment of adverse drug reactions

As shown in **Table 3**, the clinical response was evaluated in 306 patients receiving VCZ for the treatment. There was no significant difference in total favorable response (complete plus partial) between groups B and A (*p* = 0.575) or among subgroups (group C1, C2, D1, D2, E1, and E2) (*p* = 0.735).

**Table 3:** Comparisons of VCZ clinical efficacy among the groups.

Clinical efficacy	Gene-directed group (n=148)				Non-gene-directed group (A) (N=158)	Gene-directed group (B) (N=148)
	UM/RM/NM (C2) (N=64)	IM (D2) (N=68)	PM (E2) (N=16)	PM (E1) (N=18)		
Complete response (*n*, %)	43 (67.19)	42 (61.76)	12 (75.00)	15 (83.33)	105 (66.46)	97 (65.54)
Partial response (*n*, %)	9 (14.06)	16 (23.53)	2 (12.50)	2 (11.11)	31 (19.62)	27 (18.24)
Treatment failure (*n*, %)	12 (18.75)	10 (14.71)	2 (12.50)	1 (5.56)	22 (13.92)	24 (16.22)
*P*-Value ^1)^	0.564				0.575	

Data are presented as the number for categorical variables. A Chi-squared test, Compare the total favorable response (complete plus partial responses) in all groups.

**Figure 5:**
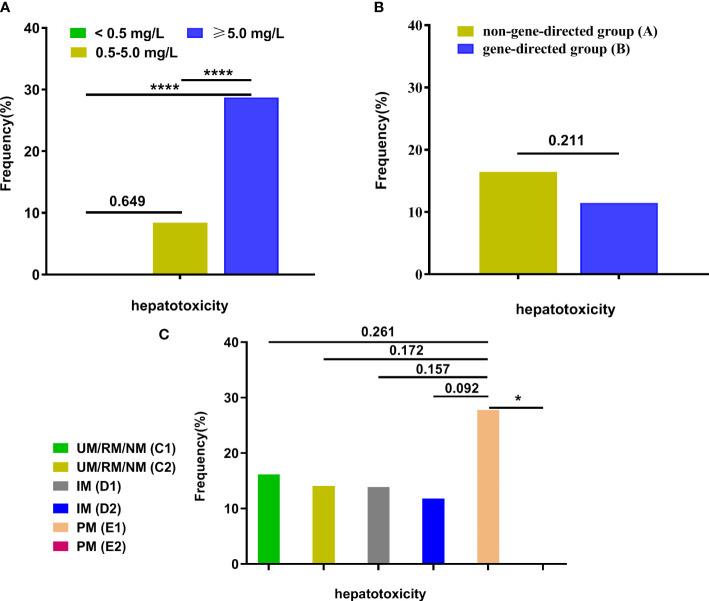
The frequency of hepatotoxicity among groups. **(A)** The frequency of hepatotoxicity among different VCZ-*C*_min_ (< 0.5, 0.5-5.0, ≥ 0.5 mg/L); **(B)** The frequency of hepatotoxicity between group A and group **(B, C)** The frequency of hepatotoxicity among subgroups (UM/RM/NM (C1), UM/RM/NM (C2), IM (D1), IM (D2), PM (E1), and PM (E2)). Data were described as their number for categorical variables and were analyzed by a Chi-squared test or Fisher’s exact test. *p<0.05; ****p<0.0001. VCZ-*C*_min_, voriconazole serum trough concentration; UM, Ultrarapid metabolizer; RM, rapid metabolizer; NM, normal metabolizer; IM, intermediate metabolizer; PM, poor metabolizer.

Out of 306 patients, 40 had hepatotoxicity. We assessed the effects of different levels of VCZ-*C*_min_ (< 0.5, 0.5-5.0, ≥ 0.5 mg/L) on the incidence of hepatotoxicity. Hepatotoxicity differed significantly among the different levels of VCZ-*C*_min_(< 0.5, 0.5-5.0, ≥ 0.5 mg/L) (χ^2 ^= 19.253, *p* < 0.0001). The incidence of hepatotoxicity was higher in the supratherapeutic range compared to the therapeutic and subtherapeutic ranges (*p* < 0.001), respectively (**Figure 5A**). There was no significant difference in the incidence of hepatotoxicity between groups A and B (*p* = 0.211) (**Figure 5B**). No significant differences in hepatotoxicity were found among subgroups (group C1, C2, D1, D2, E1, and E2, *p* = 0.309). However, the incidence of hepatotoxicity was lower in group E2 compared to group E1 (*p* = 0.031), respectively (**Figure 5C**).

### Analysis of factors affecting VCZ-*C*_min_

Factors affecting VCZ-*C*_min_ were evaluated in 302 patients using a Chi-squared test. The results showed that age, CYP2C19 phenotype (PM), CRP, PCT, IL-6, and average daily dose/body weight were risk factors for VCZ-*C*_min_ ≥ 5.0 mg/L (*p* < 0.05) (**Table 4**). The CRP, PCT, IL-6, and average daily dose/body weight values were stratified according to the cutoff values predicted by the ROC curve. A multiple linear stepwise regression analysis was performed by using VCZ-*C*_min_ (Y) as the dependent variable, and sex (x_1_), age (x_2_), body weight (x_3_), VCZ route of administration (x_4_), CYP2C19 phenotype (x_5_), the average daily dose (x_6_), combined PPIs (x_7_), combined methylprednisolone (x_8_), CRP (x_9_) and average daily dose/body weight (x_10_) as independent variables. The regression equation is Y=1.616 + 0.015x_9 _+ 0.169x_10 _+ 0.379x_5_ (*p* < 0.001). It showed that CRP, average daily dose/body weight, and CYP2C19 phenotype were significant factors influencing VCZ-*C*_min_, respectively (*p* < 0.05) (**Table 5**).

**Table 4:** Correlation between VCZ-*C*_min_ ≥ 5.0 mg/L and influencing factors.

Indexes	Layering	N (the number of VCZ-*C*_min_≥5.0 mg/L/total numbers)	P*-*Value	OR(95% CI)
Gender	Male	55/189	0.506	1.186 (0.717, 1.961)
	Female	37/113		
Age (years)	≤55	11/56	0.029	2.395 (1.071, 5.352)
	>55	81/246		
VCZ route of administration	Oral	20/80	0.216	1.440 (0.807, 2.569)
	Intravenous	72/222		
Body weight (kg)	≥60	54/186	0.494	1.191 (0.722, 1.965)
	<60	38/116		
CYP2C19 Phenotype	UM/EM/NM	34/127		
	IM	41/138	0.596	1.156 (0.676, 1.977)
	PM	17/37	0.027	2.325 (1.091, 4.954)
PPIs (esomeprazole and omeprazole)	Used	39/142	0.286	1.308 (0.798, 2.144)
	Unused	53/160		
Methylprednisolone	Used	25/90	0.509	1.201 (0.697, 2.071)
	Unused	67/212		
CRP (mg/L)	<50	31/162		
	50-100	30/76	0.001	2.756 (1.506, 5.042)
	≥100	31/64	<0.001	3.970 (2.120, 7.434)
PCT (ng/mL)	<1.00	53/204	0.002	2.442 (1.368, 4.359)
	≥1.00	30/65		
IL-6 (pg/mL)	<28.50	15/89	< 0.001	6.272 (3.065, 12.836)
	≥28.50	37/72		
Average daily dose/body weight (mg/Kg)	<7.815	40/163	0.015	1.838 (1.120, 3.016)
	≥7.815	52/139		

Data are presented as the number of categorical variables. A Chi-squared test compared categorical data. VCZ-C_min_, voriconazole serum trough concentration; PPIs, proton pump inhibitors; CRP, C-reactive protein; PCT, procalcitonin; IL-6, interleukin-6.

**Table 5:** ** **A multiple linear stepwise regression analysis factors influencing VCZ-*C*_min_.

Model	Unstandardized		Standardized Coefficients	*t*	*P-*Value
	B	Std.Error	Beta		
Constant	1.616	0.565		2.859	0.005
CRP (mg/L)	0.015	0.002	0.396	7.598	<0.001
Average daily dose/body weight (mg/Kg)	0.169	0.069	0.129	2.469	0.014
CYP2C19 phenotype (UM/RM/NM:0, IM:1, PM:2)	0.379	0.178	0.110	2.126	0.034

VCZ-C_min_, voriconazole serum trough concentration; CRP, C-reactive protein; UM, Ultrarapid metabolizer; RM, rapid metabolizer; NM, normal metabolizer; IM, intermediate metabolizer; PM, poor metabolizer.

### Analysis of factors affecting VCZ maintenance dose

A multiple linear stepwise regression analysis was performed using VCZ stable maintenance doses (Y) as a dependent variable and sex (x_1_), age (x_2_), VCZ route of administration (x_3_), body weight (x_4_), CYP2C19 phenotype (x_5_), combined PPIs (x_6_), combined glucocorticoid (x_7_), CRP (x_8_) as independent variables. The regression equation is Y=360.126-30.325x_5_ -0.215x_8 _+ 0.888x_4_ (*p* < 0.001). The results showed that the CYP2C19 phenotype, CRP, and body weight were significant factors influencing VCZ maintenance dose, respectively (*p* ≤ 0.05) (**Table 6**). Although age also affected the stable maintenance dose of VCZ, it was not significant (*p* = 0.242).

**Table 6:** ** **A multiple linear stepwise regression analysis factors influencing VCZ maintenance dose.

Model	Unstandardized		Standardized Coefficients	*t*	*P-*Value
	B	Std.Error	Beta		
Constant	360.126	25.096		14.350	<0.001
CYP2C19 gene phenotype (UM/EM/NM:0, IM:1, PM:2)	-30.325	6.493	-0.257	4.670	<0.001
CRP (mg/L)	-0.215	0.072	-0.165	3.0013	0.003
Body weight (Kg)	0.888	0.383	0.128	2.317	0.021

VCZ, voriconazole; CRP, C-reactive protein; UM, Ultrarapid metabolizer; RM, rapid metabolizer; NM, normal metabolizer; IM, intermediate metabolizer; PM, poor metabolizer.

### Establishment and validation of prediction model for VCZ stable maintenance dose

According to the admission order, the 302 patients were divided into the derivation and validation groups. There were no significant differences in the general clinical data (sex, age, body weight, albumin, co-administered PPIs, co-administered hormones, CYP2C19 phenotype, underlying disease, etc.) between the derivation and validation groups (*p* > 0.05).

In the derivation group, the stable maintenance dose was used as the independent variable (Y), and age (x_1_), body weight (x_2_), CYP2C19 phenotype (x_3_), and CRP (x_4_) were the dependent variables. The predictive model is Y=282.774-0.735x_1 _+ 2.946x_2_-19.402x_3_-0.316x_4_, *p* < 0.001. To make the precise dose predicted by the model more convenient for clinicians and nurses, the predicted doses of 175~224, 225~274, 275~324, 325~374, 375~424, 425~474, 475~524, 525~574, and 575~624 mg were adjusted to 200, 250, 300, 350, 400, 450, 500, 550, and 600 mg, respectively.

There was no significant difference between the predicted VCZ maintenance dose and the actual maintenance dose according to the predictive model and the actual maintenance dose (376.10 ± 57.87 vs. 374.00 ± 47.93 mg, *p* = 0.686). The difference rate (20%~50% and ≥ 50%) between the predicted dose and actual stable dose was 12.00% (12/100) and 1.00% (1/100), respectively.

The data of 13 patients with different rates (≥ 20%) were compared and analyzed. It was shown that the *C*_min_ of 6 patients (5.34 mg/L ≤ *C*_min_ ≤ 10.17 mg/L) was ≥ 5.0 mg/L, and 7 patients (1.35 mg/L ≤ *C*_min_ ≤ 4.17 mg/L) were in the range of 0.5-5.0 mg/L. The essential daily maintenance was achieved when *C*_min_ was in the 0.5-5.0 mg/L range by adjusting the dosage of VCZ. Subsequently, the maintenance dosage predicted by the model was used for the 13 patients, and VCZ-*C*_min_ was measured 5 days later. It was shown that the VCZ-*C*_min_ of 11 patients was in the range of 0.5-5.0 mg/L, except for two patients (5.06 mg/L and 5.39 mg/L). This means that if 100 patients received maintenance doses according to the predictive model, the VCZ-*C*_min_ for 98% of all patients would be in the 0.5-5.0 mg/L range. Because the target VCZ-*C*_min_ is a range, even if there is a deviation of more than 20% between the predicted stable maintenance dose and the maintenance dose initially adjusted to the target *C*_min_, the VCZ-*C*_min_ will ultimately be in the 0.5-5.0 mg/L range by using the dose of VCZ according to the predictive model.

## Discussion

In many studies, most CYP2C19 mutations involved three common alleles in the Chinese Han population, namely CYP2C19 *2,*3, and *17 (Chow et al., 2019). The current study was conducted on the Henan Chinese Han patient population, and the allele frequencies for CYP2C19 *2,*3, and *17 were found to be 30.8%, 3.8%, and 1.3%, respectively. A Chi-squared test revealed that the distribution of each genotype was consistent with the Hardy-Weinberg law. These allele frequencies were similar to those reported by Botton MR et al. in Asians (28%, 7%, and 2%) and by Zuo LJ et al. in Chinese populations (50.0%, 6.3%, and 2.1%) (Zuo et al., 2012; Botton et al., 2021). It is well known that genetic polymorphism of CYP2C19 can cause phenotypic variability (Chen et al., 2022), contributing to the high variability of VCZ exposure, affecting drug efficacy, and leading to ADRs.

Due to the high bioavailability of oral dosage form (96%) according to VCZ’s label, the intravenous and oral routes of administration are interchangeable when clinically indicated. In the study, 306 patients were treated with different routes of administration for VCZ (Intravenous, oral and sequential therapy). The results showed no noticeable difference in different routes of administration between groups A and B (*p >* 0.05). Additionally, there was no significant difference in VCZ-*C*_min_ among the three routes of administration (*p* > 0.05). Our findings highlight the role that CYP2C19 phenotype may have in managing IFI patients with low-dose VCZ in PMs. Furthermore, no relationship between VCZ efficacy and CYP2C19 status was found, consistent with Wang T et al.’s study (Wang et al., 2014). The study also found that CYP2C19 gene-directed administration has an excellent guiding effect on the use of VCZ and reduces the occurrence of hepatotoxicity, especially for patients with PM phenotype. CYP2C19 phenotype is a risk factor affecting VCZ-*C*_min_ and plays a critical role in the initial loading dose of VCZ.

Although CRP and IL-6 are known to be involved in the high fluctuation of VCZ-*C*_min_ (Encalada Ventura et al., 2016; Bolcato et al., 2021), little is known about the influence of PCT on it. We hypothesize that increased inflammatory factors will increase the risk of high VCZ-*C*_min_ and may increase VCZ-related toxicity during treatment. This is the first study to show that inflammation, as reflected by CRP, PCT, and IL-6, can independently influence VCZ-*C*_min_, completely masking the effects of other potential risk factors. In our study, by measuring CRP, PCT, and IL-6 levels at the different therapeutic ranges, we can determine their potential contribution to the upregulation of VCZ-*C*_min_. CRP, PCT, and IL-6 in the supratherapeutic range are significantly higher than in other treatment ranges. Our study found that inflammation, as assessed by CRP level, is a risk factor affecting VCZ-*C*_min_ by Stepwise regression analysis. This finding is consistent with Van Wanrooy MJ et al. (Van Wanrooy et al., 2014). In our study, for every 1mg/L increase in the CRP concentration, the VCZ-*C*_min_ was 0.015 mg/L higher (VCZ-*C*_min_ (mg/L)=1.616 + 0.379×CYP2C19 phenotype+0.015×CRP (mg/L)+0.169×average daily dose/body weight (mg/Kg)) (*p* < 0.001). To further clarify this finding, we analyzed the data changes of VCZ-*C*_min_ accompanied by inflammatory factors measured at different times in the same patient. The results showed that VCZ-*C*_min_ changes more significantly along with the inflammatory factors in the same direction.

These findings indicate that high inflammation, reflected by CRP, PCT, and IL-6, can affect the pharmacokinetics of VCZ, resulting in an increase in *C*_min_ in patients. This is probably related to inflammation-induced phenoconversion (Shah and Smith, 2015), where elevated amounts of inflammatory cytokines can cause down-regulation of cytochrome P450 isoenzymes at the level of gene transcription, resulting in a decrease in corresponding mRNA, protein, and enzyme activities. Inflammation stimulates the release of cytokines, which can modulate the liver’s transcription factor activities. As a result, the metabolism of VCZ metabolized by CYP2C19 decreases, causing a greater risk of overdose, and hence the VCZ-*C*_min_ increases (Shah and Smith, 2015; Darakjian et al., 2021). We also find that PCT influences VCZ-*C*_min_. During inflammation, PCT production is increased by stimulating IL-1, IL-6, and TNF-α, which can down-regulate expression enzymes and change the patient’s metabolic phenotype. Our study indicates that VCZ-*C*_min_ is more likely to exceed 5.0 mg/L when PCT is ≥ 0.995 ng/mL (*p* < 0.001), suggesting that these patients may be more likely to suffer from VCZ toxicity. These findings are consistent with those of Zeng G et al. (Zeng et al., 2020).

Our study found that a multiple linear stepwise regression did not identify PPIs or glucocorticoids as significant covariates for VCZ exposure. These covariates may be due to multiple factors, such as high inflammation and poor metabolism genotype, that could mask the results of drug interactions. Previous studies have shown that VCZ exposure increases with the type of PPI used (Blanco Dorado et al., 2020), but the influence of glucocorticoids on VCZ exposure remains controversial (Li et al., 2017; Li et al., 2018).

Although CPIC^®^ guidelines for VCZ treatment specify therapy and drug alternatives for VCZ based on CYP2C19, other factors affecting VCZ-*C*_min_ have not been considered. In order to achieve the steady-state target VCZ-*C*_min_ quickly, the present study was performed to develop a mathematical model of the VCZ maintenance dose for IFIs adult patients with normal liver function based on multiple risk factors, including the CYP2C19 phenotype, CRP, body weight, and age. This model aims to provide an individualized treatment strategy for VCZ. During periods of high variation in inflammatory factors, the VCZ maintenance dose can be adjusted to reach the target *C*_min_ at any time using the mathematical model. Our study found that satisfactory clinical efficacy can also be achieved when the standard or increased dosing is administered for patients with UM and RM based on multiple factors. The mathematical model developed in this study may be a valuable supplement to CPIC^®^ guidelines for VCZ treatment. We included CRP instead of PCT and IL-6 in the prediction model because some patients lack data on PCT and IL-6. CRP concentrations are widely used as markers for inflammation in daily practice.

In addition to the significant covariates that affect the VCZ maintenance dose mentioned above, several other covariates must be considered. Liver dysfunction affects the clearance of VCZ, and patients with abnormal liver function often experience increased VCZ exposure due to slowed clearance when using conventional doses. The established model is based on patients with normal liver function without considering the effect of liver injury on the dose. Therefore, for patients with abnormal liver function, the dose should be appropriately reduced based on the recommended dose of the model during clinical treatment with VCZ. For individual patients whose VCZ maintenance dose is less than 200 mg/d or more than 600 mg/d, a significant deviation from the predicted dose appears in the established prediction model. There may be some other influencing factors that still need to be discovered.

## Conclusions

Our study provides evidence of the variability of inflammatory factors affecting VCZ-*C*_min_. Consequently, physicians should be aware of this phenomenon to avoid the toxicity caused by increased VCZ concentration due to high inflammation. Based on these significant factors, we have also established a predictive model of the VCZ stable maintenance dose. Proper stable maintenance administration of the predictive model can help patients quickly reach the target *C*_min_. This model may be a valuable supplement to CPIC^®^ guidelines for VCZ treatment, and awareness of it may improve the benefit/risk ratio of the drug.

## Data availability statement

The original contributions presented in the study are included in the article/**Supplementary Materials**, further inquiries can be directed to the corresponding authors.

## Ethics statement

The studies involving human participants were reviewed and approved by The Investigational Review Board at Zhengzhou Central Hospital Affiliated to Zhengzhou University. The patients/participants provided their written informed consent to participate in this study. Written informed consent was obtained from the individual(s) for the publication of any potentially identifiable images or data included in this article.

## Author contributions

LZ, LC, and QX designed the research, analyzed and interpreted the data, performed the statistical analysis, drafted the manuscript, and incorporated feedback from all authors. ML and HL coordinated and managed study data acquisition. LZ, ZS, and HL examined all the specimens and collected clinical data. ZG, YG, HZ, and FQ collected clinical specimens, supervised data acquisition, and analyzed and interpreted data. WC co-conceptualized the study, designed the research, and revised the manuscript. All authors contributed to the article and approved the submitted version.

## Supplementary material

Supplementary Figure 1
**(A-AB)** The values of VCZ-*C*_min_ (mg/L), CRP (mg/L), PCT (ng/mL) and IL-6 (pg/mL) of each patient at different time points. **A–AB** represent different patients, respectively. The first, second, third, and fourth time points are 3-5 days, 7-10 days, 11-14 days, and 16-20 days after VCZ administration. VCZ-*C*_min_, voriconazole serum trough concentration; CRP, C-reactive protein; PCT, procalcitonin; IL-6, interleukin-6.
