# Supplementary figures and images for: Establishment of a mathematical prediction model for voriconazole stable maintenance dose: a prospective study
Source: Front Cell Infect Microbiol. 2023 Jul 26;13:1157944. doi: 10.3389/fcimb.2023.1157944 (PMC10410275; doi:10.3389/fcimb.2023.1157944)

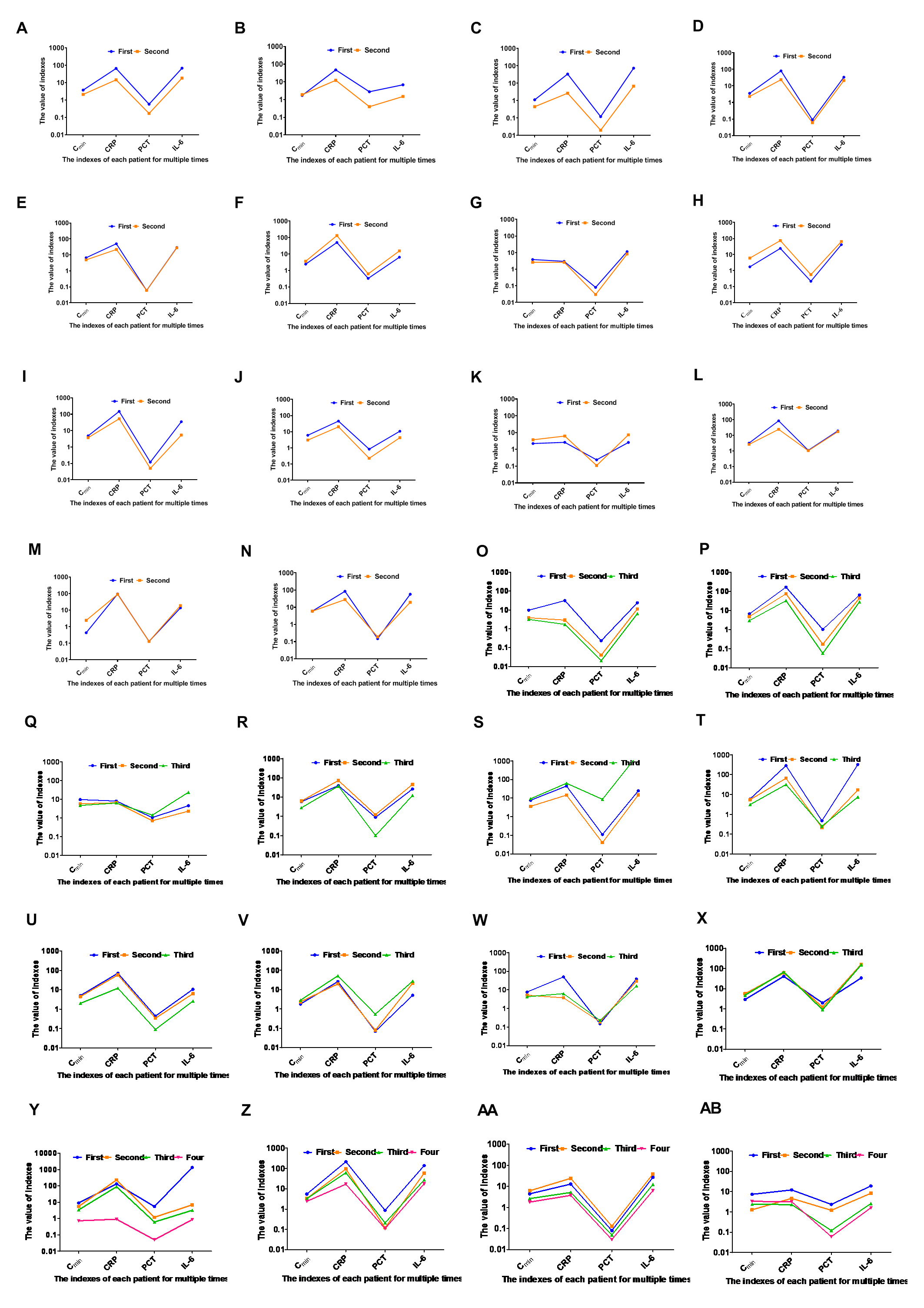

Supplement: Supplementary Figure 1 — (A-AB) The values of VCZ-C min (mg/L), CRP (mg/L), PCT (ng/mL) and IL-6 (pg/mL) of each patient at different time points. A–AB represent different patients, respectively. The first, second, third, and fourth time points are 3-5 days, 7-10 days, 11-14 days, and 16-20 days after VCZ administration. VCZ-C min, voriconazole serum trough concentration; CRP, C-reactive protein; PCT, procalcitonin; IL-6, interleukin-6. [file Image_1.tif]
